# Dicaffeoylquinic acid alleviates alcoholic liver disease by targeting PLA2G4B and inhibiting the MAPK signaling pathway

**DOI:** 10.3389/fphar.2026.1823992

**Published:** 2026-05-29

**Authors:** Linlin Wang, Hailong Wang, Yu Miao, Hamulati Hasimu, Tengfei Ji, Hua Huang, Guanhua Du, Haji Akber Aisa, Xuelei Xin

**Affiliations:** 1 State Key Laboratory Basis of Xinjiang Indigenous Medicinal Plants Resource Utilization, and the Key Laboratory of Chemistry of Plant Resources in Arid Regions Xinjiang Technical Institute of Physics and Chemistry, Chinese Academy of Sciences, Urumqi, China; 2 Xinjiang Key Laboratory of Uygur Medicine, Xinjiang Institute of Materia Medica, Urumqi, China; 3 University of Chinese Academy of Sciences, Beijing, China; 4 Xinjiang Medical University, Urumqi, China; 5 State Key Laboratory of Bioactive Substance and Function of Natural Medicines, Institute of Materia Medica, Chinese Academy of Medical Sciences and Peking Union Medical College and Beijing Key Laboratory of Drug Target and Screening Research, Beijing, China

**Keywords:** 4,5-dicaffeoylquinic acid, alcoholic liver disease, *Artemisia scoparia*, MAPK signaling pathway, PLA2G4B

## Abstract

**Introduction:**

Alcoholic liver disease (ALD) is a serious global health burden with limited effective therapies. 4,5-Dicaffeoylquinic acid (DCA-C), a monomer from Artemisia scoparia, exhibits multiple bioactivities, but its protective effects and exact mechanisms against ALD remain unclear.

**Methods:**

Ethanol-induced ALD mouse models were treated with DCA-C at 10, 30, and 100 mg/kg. Liver injury, lipid accumulation, and inflammation were evaluated. Transcriptomic sequencing and molecular docking were used to identify key targets. PLA2G4B overexpression models in vitro and in vivo were constructed to validate the mechanism.

**Results:**

DCA-C dose-dependently alleviated alcoholic liver injury, with the 100 mg/kg dose being most effective, as shown by reduced serum ALT/AST, decreased hepatic triglycerides, and suppressed pro-inflammatory cytokines. PLA2G4B was identified as a core target of DCA-C. DCA-C directly bound PLA2G4B, downregulated its expression, and reduced arachidonic acid (AA) release, thereby inhibiting MAPK signaling (ERK, p38, JNK phosphorylation). PLA2G4B overexpression abolished the protective effects of DCA-C *in vitro* and *in vivo*.

**Discussion:**

DCA-C protects against ALD via targeting PLA2G4B and inhibiting the AA-MAPK pathway. DCA-C is a promising candidate for ALD treatment and warrants further development.

## Graphical Abstract

**Figure:**
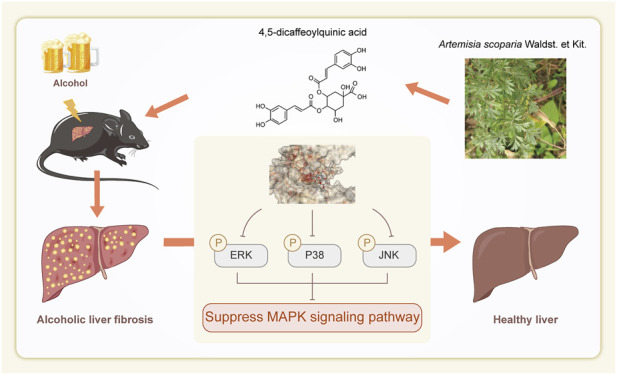

## 1 Introduction

Alcoholic liver disease (ALD), a spectrum of liver injuries induced by long-term excessive alcohol consumption, has emerged as one of the leading causes of liver-related morbidity and mortality worldwide (Wu et al., 2023; Mackowiak et al., 2024). Notably, ALD accounts for over 25% of liver-associated deaths globally (Asrani et al., 2019). The pathogenesis of ALD involves a complex cascade of pathological events. Ethanol metabolism disrupts hepatic lipid homeostasis by promoting lipogenesis and inhibiting fatty acid oxidation, leading to steatosis. Ethanol oxidation generates excessive reactive oxygen species and acetaldehyde, which collectively trigger oxidative stress, mitochondrial dysfunction, and direct hepatocellular damage. Injured cells release damage-associated molecular patterns (DAMPs), initiating sterile inflammation (Liu et al., 2021; Silitonga et al., 2024). Moreover, chronic alcohol consumption increases intestinal permeability and promotes the accumulation of gut-derived endotoxins, facilitating the translocation of lipopolysaccharide (LPS) from the intestine to the liver. LPS binds to Toll-like receptor 4 (TLR4) on the surface of Kupffer cells, activating both the MyD88-dependent pathway, which mediates NF-κB signaling, and the MyD88-independent pathway involving interferon regulatory factor 3 (IRF3), triggering the synthesis and release of cytokines and inflammatory mediators, which in turn stimulates the recruitment and accumulation of neutrophils and macrophages, ultimately exacerbating hepatic inflammation and liver damage (Gao et al., 2019; Bataller et al., 2022). Beyond direct hepatocyte injury, chronic alcohol exposure profoundly impairs hepatic phospholipid metabolism. Ethanol consumption reduces hepatic phosphatidylcholine (PC) levels while increasing phosphatidylethanolamine (PE) levels, leading to a decreased PC/PE ratio (Cao et al., 2022), the PC/PE ratio influences the structural integrity of hepatocyte membranes, further exacerbating hepatic steatosis. Despite extensive research efforts over the past decades, effective therapeutic strategies for ALD remain scarce. The clinical management of ALD is mainly based on lifestyle interventions such as alcohol abstinence and nutritional support, which can only slow down the progression of the disease but cannot reverse existing liver damage (Tadokoro et al., 2023). Moreover, there are currently no FDA-approved drugs available for clinical application (Park et al., 2022). For these reasons, natural products have emerged as potential therapeutic alternatives, owing to their multi-component properties and capacity to modulate multiple biological targets simultaneously.

4,5-Dicaffeoylquinic acid (DCA-C) is a monomer with diverse biological activities such as antioxidative, anti-inflammatory, hypoglycemic, cardiovascular and hepatoprotective effects. These properties have been well-documented in modern studies (Jang et al., 2022; Hufnagel et al., 2024; Huang et al., 2025). In terms of anti-inflammatory effects, DCA-C can inhibit the NF-κB and MAPK pathways, thereby reducing the release of pro-inflammatory factors such as TNF-α (Jang et al., 2021). For antioxidant activity, it exerts protective effects through free radical scavenging and reducing power, which helps mitigate oxidative stress-induced cellular damage. In mitochondrial regulation, it possesses cytochrome c reductase activity and modulates mitochondrial function in a dose-dependent manner (Kamarauskaite et al., 2021). Additionally, a previous study indicated that DCA-C modulates glucose and cholesterol metabolism. It also improves metabolic homeostasis by regulating the expression of PKC-α, SREBP-1, and FAS, while inhibiting the activation of the polyol pathway (Tseng et al., 2025). In our previous experiments, we demonstrated that DCA-C exerts a protective effect against CCL_4_-induced liver injury. Importantly, DCA-C has demonstrated anti-inflammatory, antioxidant, and lipid-regulating activities in various disease models. ALD is pathologically characterized by steatosis, inflammation, and oxidative stress, DCA-C may therefore exert protective effects against ALD. However, current evidence on the role of DCA-C in ALD remains insufficient, and the specific molecular mechanisms underlying its effects on ALD pathological progression have yet to be elucidated. Therefore, further investigation into the therapeutic effects of DCA-C on ALD and its associated molecular mechanisms is essential to fill this research gap.

PLA2G4B is a member of the A2 family of cellular membrane phospholipases that selectively hydrolyzes glycerophospholipids at the sn-2 position, with a preference for arachidonic acid acyl phospholipids (Gao et al., 2021). It has been reported that PLA2G4B inhibits the breakdown of phosphatidylcholine (PC), thereby reducing the production of its derived lipid mediators and slowing tumor progression (Recuero et al., 2024). Additionally, downregulation of PLA2G4B has been involved in phosphatidylcholine conversion to anti-inflammatory lipoxins (Saare et al., 2020). Given the central roles of lipid dysregulation and inflammatory signaling in the pathogenesis of ALD, we hypothesize that PLA2G4B may critically regulate disease progression. However, the specific role of PLA2G4B in ALD remains poorly understood, and its regulatory mechanisms warrant further investigation.

Our study showed that DCA-C protects against ALD in both *in vitro* and *in vivo* models. This is achieved by targeting PLA2G4B and inhibiting the MAPK signaling pathway, thereby alleviating liver injury, lipid accumulation and inflammation.

## 2 Materials and methods

### 2.1 Reagents and antibodies

DCA-C (CAS: 57378-72-0) was purchased from Manster Biotechnology (Chengdu, China). Bicyclol was supplied by Institute of Materia Medica, Chinese Academy of Medical Sciences and Peking Union Medical College, Liquid Diet containing (by weight) 56% fat, 27% protein, 17% carbohydrates (L10016A), and 36% fat, 17% protein, 47% carbohydrates (L10015A) were supplied by Readydietech (Shenzhen) Co., Ltd., ALT (#20230314), AST (#20230308), TG (#020090) and TC (#020080) were supplied by Biosino Bio-Technology and Science Inc (Beijing, China). Lipofectamine 3000 (#L3000001, Invitrogen). Primary antibodies were as follows: ERK (#4695, Cell Signaling Technology), p-ERK (#4370, Cell Signaling Technology), JNK (#9252, Cell Signaling Technology), p-JNK (#9251, Cell Signaling Technology), p38 (#14064-1-AP, Proteintech), p-p38 (#28796-1-AP, Proteintech), and PLA2G4B (#PA5-98062, Thermo Fisher Scientific). AA (Arachidonic Acid) ELISA Kit: Elabscience (E-EL-0051).

### 2.2 Animal experiment

Male C57BL/6J mice (6–8 weeks) were procured from Beijing HFK Biotechnology Co., Ltd (License No. SYXK (Jing) 2019-0023). Animal experiments were approved by the Laboratory Animal Welfare Ethics Committee of Xinjiang Institute of Materia Medica (Registration number: XJIMM-20230611). Mice were maintained under specific pathogen-free (SPF) conditions with *ad libitum* access to food and water, the housing environment was controlled at 21 °C–25 °C with a 12-h light/dark cycle. To induce alcoholic liver injury, the study employed the well-established National Institute on Alcohol Abuse and Alcoholism (NIAAA) model, which involves chronic ethanol feeding combined with multiple binges ethanol administration (Lamas-Paz et al., 2018). Briefly, for the first five days, all groups were given a control liquid Lieber-DeCarli diet to help them adjust. The mice were randomly assigned to different groups: Control group, Model group, Positive control group (Bicyclol, 200 mg/kg), and DCA-C treatment groups at low (10 mg/kg), medium (30 mg/kg), and high (100 mg/kg) doses, with 10 mice in each group. For the following 10 days, the ethanol model group and all treatment groups were fed an ethanol-containing liquid diet (L10016A, containing 5% vol/vol ethanol, Trophic), and to facilitate adaptation, the ethanol concentration was gradually increased from 1% to 5%. The control diet group received an isocaloric control liquid diet (L10015) throughout the experiment. All animals had *ad libitum* access to their respective liquid diets, which were freshly prepared and replaced daily. Drugs (or vehicle) were administered daily by oral gavage starting from the first day of ethanol feeding. Specifically, the positive control group received bicyclol (200 mg/kg), the three treatment groups received DCA-C at low (10 mg/kg), medium (30 mg/kg), or high (100 mg/kg) doses, while the control group and ethanol model group received an equal volume of 0.5% CMC-Na vehicle. On day 11, after a 9-h fast, mice in the ethanol-fed groups received a single oral gavage of ethanol (31.5% vol/vol, 20 mL/kg), while the control diet group received an isocaloric maltodextrin solution (45% vol/vol, 20 mL/kg). After 9 h, mice were anesthetized with isoflurane. Blood samples were collected via orbital bleeding, and serum was separated by centrifugation. Liver tissues were rapidly excised, weighed, and either snap-frozen in liquid nitrogen or fixed in 4% paraformaldehyde for subsequent analysis. Body weights were recorded weekly throughout the study period. The liver index was calculated as the ratio of organ weight (mg) to body weight (g).

To investigate the role of the PLA2G4B-MAPK pathway in mediating the protective effects of DCA-C against ALD *in vivo*, male C57BL/6J mice were divided into five groups (n = 5 per group): Control, Model, DCA-C treatment, OE-NC (empty vector control), and OE-PLA2G4B. Following the induction of ALD and DCA-C treatment, mice in the OE-NC and OE-PLA2G4B groups further received tail vein injections of AAV8 carrying either the empty vector or the PLA2G4B overexpression construct (Fang et al., 2018).

### 2.3 Liver function biochemical indicators

Serum was collected after centrifuging the blood samples. Total protein concentration in the supernatant was determined using a BCA assay kit (Beyotime, China). Levels of total cholesterol (TC), aspartate aminotransferase (AST), and alanine aminotransferase (ALT), as well as triglycerides (TG) in serum, were measured using commercial kits according to the manufacturer’s protocols.

### 2.4 Histological staining

For histopathological evaluation, liver samples were fixed in 4% paraformaldehyde, processed through graded ethanol and xylene, and embedded in paraffin. Sections (5 μm) were then stained with H&E per the manufacturer’s protocol (Solarbio, China). For lipid visualization, fresh tissues were embedded in OCT compound, cryosectioned at 6 μm, and stained with Oil Red O (Beyotime, China) followed by appropriate counterstaining.

### 2.5 Cell culture

The human normal liver cell line LO2 was acquired from the Cell Bank of the Chinese Academy of Sciences (Shanghai, China) and maintained in Dulbecco’s Modified Eagle Medium (DMEM, Procell) supplemented with 10% FBS (Procell) and 1% penicillin-streptomycin (Procell). Cells were cultured at 37 °C in a humidified atmosphere of 95% air and 5% CO_2_.

Primary mouse hepatocytes were isolated using a two-step *in situ* collagenase perfusion method. Briefly, the liver was first perfused with calcium-free HBSS to remove blood, followed by perfusion with collagenase IV-containing digestion buffer. Hepatocytes were filtered, purified by low-speed centrifugation, and cultured on collagen-coated plates at 37 °C with 5% CO_2_ (Feng et al., 2021).

Cells were categorized into six groups: a control group, a model group, three groups treated with different concentrations of DCA-C, and a positive drug group. An *in vitro* ALD model was established by stimulating cells with 100 mM alcohol for 24 consecutive hours (Qi et al., 2022). Meanwhile, cells in the DCA-C-treated groups and Bicyclol group were cultured in medium containing 100 mM alcohol, with the addition of DCA-C-containing serum or positive drug, respectively, for the same duration.

For PLA2G4B overexpression, cells were transfected with OE-PLA2G4B plasmid or the corresponding empty vector using Lipofectamine 3000 transfection reagent (Invitrogen) according to the manufacturer’s instructions.

### 2.6 Quantitative real-time PCR

Total RNA was extracted from tissues or cultured cells using TRIzol reagent (Invitrogen). RNA concentration and purity were determined spectrophotometrically. Subsequently, 1 µg of total RNA was reverse-transcribed into cDNA using a PrimeScript RT reagent kit (Takara). Quantitative PCR was then performed in triplicate using SuperReal PreMix Plus (TIANGEN). The relative mRNA expression levels were calculated using the 2^−ΔΔCt^ method, with GAPDH serving as the internal control. The primer sequences for mouse and human hepatocytes are as follows (5'→3′): PLA2G4B (mouse): GTC​TCA​TGG​CTC​TGC​AAA​CCT (Forward), TTG​ACT​GTG​CGT​GTC​TGG​AG (Reverse). PLA2G4B (human): ACT​CAG​TCT​CAT​GGC​TGT​GG (Forward), TCA​GAG​GGG​GTC​ACT​AGG​TC (Reverse). GAPDH (mouse): CAA​CTA​CAT​GGT​CTA​CAT​GTT​C (Forward), CGC​CAG​TAG​ACT​CCA​CGA​C (Reverse). GAPDH (human): GTC​TCC​TCT​GAC​TTC​AAC​AGC​G (Forward), ACC​ACC​CTG​TTG​CTG​TAG​CCA​A (Reverse).

### 2.7 Cellular thermal shift assay

A cellular thermal shift assay (CETSA) was performed as previously described with minor modifications. Briefly, cells were incubated with DMSO (control) or DCA-C for 24 h. Cells were then collected, washed, and resuspended in PBS with protease inhibitors. Aliquots were heated at a gradient of temperatures (37 °C–67 °C) for 5 min in a PCR thermal cycler. After cooling, cells were lysed by 3-5 repeated freeze–thaw cycles in liquid nitrogen in 0.5% NP-40 lysis buffer. Soluble proteins were separated by centrifugation (20,000 × g, 20 min, 4 °C) and analyzed by Western blotting (Yang et al., 2025).

### 2.8 Western blot

Protein extraction was performed using RIPA lysis buffer (Beyotime, China) supplemented with a protease and phosphatase inhibitor cocktail. The concentration of the extracted proteins was determined with a BCA protein assay kit (Beyotime, China). For immunoblotting, equal protein quantities were resolved by SDS-PAGE and transferred to PVDF membranes (Millipore, USA). To minimize non-specific background, membranes were blocked with 5% non-fat milk for 1 h at room temperature before being probed with the indicated primary antibodies at 4 °C overnight. Following incubation with appropriate HRP-linked secondary antibodies, protein bands were detected using an enhanced chemiluminescence (ECL) kit. GAPDH was used to normalize protein loading.

### 2.9 Immunofluorescence (IF)

Cells grown on coverslips were fixed using 4% paraformaldehyde for 15 min, followed by permeabilization with 0.1% Triton X-100 for 10 min at room temperature. After blocking with 5% BSA for 1 h, samples were incubated with primary antibody: PLA2G4B (1:100, Thermo Fisher Scientific) overnight at 4 °C. The following day, the cells were stained with a fluorophore-conjugated secondary antibody (Thermo Fisher Scientific) for 1 h at room temperature in the dark. Nuclei were counterstained with DAPI. Finally, the coverslips were mounted onto glass slides, and images were captured using a fluorescence or confocal microscope.

### 2.10 Statistical analysis

Statistical significance was determined using GraphPad Prism 9.0. All data are expressed as mean ± SD, and the experiments were repeated at least three times. We employed the Student’s t-test and one-way analysis of variance (ANOVA) for inter-group comparisons, considering results with P < 0.05 as statistically significant.

## 3 Results

### 3.1 DCA-C alleviates alcohol-induced liver injury and lipid accumulation

**FIGURE 1:**
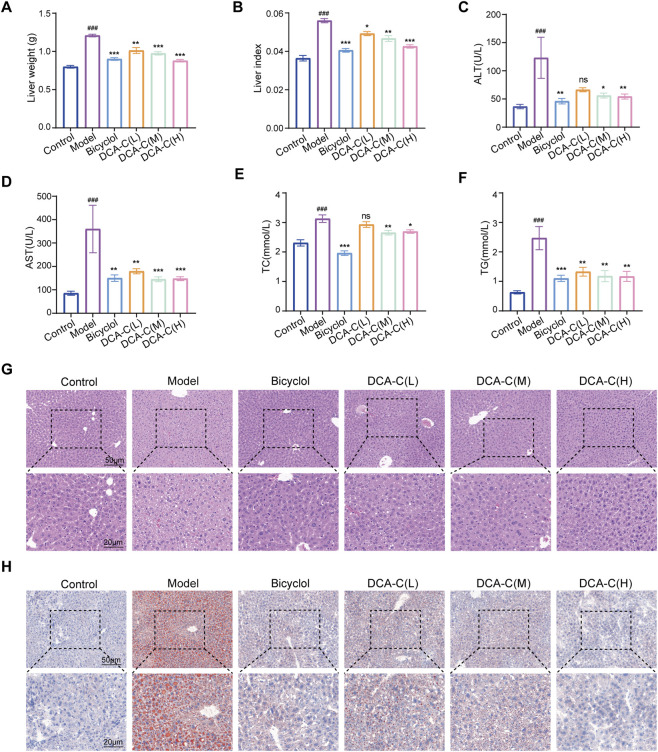
DCA-C ameliorates alcohol-induced liver injury and hepatic steatosis in mice. **(A,B)** Liver weight **(A)** and liver index (liver-to-body weight ratio) **(B)** in different groups. **(C–F)** Serum levels of ALT, AST, TC, and TG. **(G)** Representative images of hematoxylin and eosin (H&E) staining of the liver tissue, scale bars: 50 μm (upper panels), scale bars: 20 μm (lower panels). **(H)** Representative Oil Red O staining of the liver tissue, scale bars: 50 μm (upper panels), scale bars: 20 μm (lower panels). Data are presented as mean ± SD (n = 10). ^###^ *p <* 0.001 vs. Control group. *p < 0.05, **p < 0.01, ***p < 0.001 vs Model group.

To assess the therapeutic potential of DCA-C on ALD, we established an alcohol-induced mouse model and treated them with DCA-C. Alcohol-induced hepatic steatosis was assessed via serum and liver biomarkers. Compared with controls, the model group showed marked elevations in absolute liver weight (Figure 1A) and liver index (Figure 1B), indicating pathological liver enlargement. These changes were effectively improved by DCA-C treatment in a dose-dependent manner. Serum biochemical analysis demonstrated that the model group showed significant increases in serum levels of AST, ALT and TC. In contrast, DCA-C treatment resulted in a marked dose-dependent amelioration of these indicators, with the high-dose group exhibiting effects comparable to those of the positive control drug (Bicyclol, a well-known and recognized hepatoprotective agent) (Figures 1C–E). Similarly, hepatic TG accumulation was also significantly ameliorated by DCA-C treatment (Figure 1F). DCA-C treatment dose-dependently attenuated the histopathological signs of liver injury and lipid accumulation, with medium and high doses showing the most significant protective effects (Figures 1G,H). These results demonstrate that DCA-C effectively mitigates alcohol-induced liver injury along with concomitant hepatic steatosis.

### 3.2 PLA2G4B is associated with the pathogenesis of ALD

**FIGURE 2:**
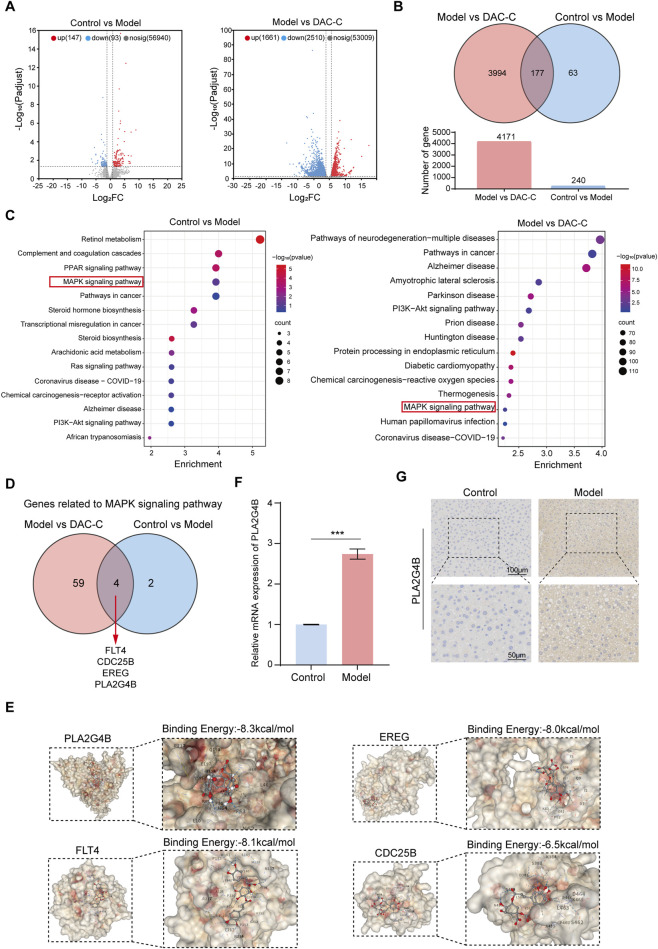
PLA2G4B is associated with ALD. **(A)** Volcano plots displaying the differentially expressed genes (DEGs) from the comparisons of Model vs. Control (left) and DCA-C-treated vs. Model groups (right). **(B)** Venn diagram showing the intersection of DEGs from the two comparisons, identifying 177 common genes. **(C)** KEGG pathway enrichment analysis of the DEG sets from the Model vs. Control and DCA-C-treated vs. Model comparisons. **(D)** Venn diagram of the MAPK signaling pathway-related DEGs, identifying four intersecting genes: FLT4, CDC25B, EREG, and PLA2G4B. **(E)** Molecular docking models of DCA-C with the four candidate proteins. **(F)** mRNA expression levels of PLA2G4B in mouse liver tissues as determined by qPCR. **(G)** Representative immunohistochemistry (IHC) images showing PLA2G4B protein expression in liver tissues from different groups, scale bars: 100 μm (upper panels), scale bars: 50 μm (lower panels). Data are presented as mean ± SD (n = 3). *p < 0.05, **p < 0.01, ***p < 0.001vs Model group.

To explore the pivotal targets and signaling pathways by which DCA-C regulates ALD, we conducted transcriptomic sequencing to identify differentially expressed genes (DEGs) by comparing the model group versus the control group, and the DCA-C-treated group versus the model group (Figure 2A). Venn diagram analysis identified 177 overlapping DEGs between the two comparison sets (Figure 2B), and KEGG enrichment analysis indicated that the MAPK pathway was significantly enriched in both DEG sets, this finding implicates the pathway in ALD progression and the modulatory role of DCA-C (Figure 2C). Further intersection analysis of MAPK pathway-related DEGs yielded four core genes, including FLT4, CDC25B, EREG, PLA2G4B (Figure 2D), and molecular docking using Auto Dock Vina revealed that PLA2G4B exhibited the highest binding affinity with DCA-C, with a binding energy of −8.3 kcal/mol (Figure 2E). A cellular thermal shift assay (CETSA) was employed to substantiate the direct engagement of DCA-C with PLA2G4B. The results demonstrated that under heat stress conditions, DCA-C markedly increased the thermal stability of PLA2G4B (Supplementary Figure S1). To validate the transcriptomic sequencing and molecular docking results, qPCR and immunohistochemistry were performed. These results confirmed that the ALD model group exhibited significant upregulation of PLA2G4B mRNA and protein expression, compared with the control group (Figures 2F,G), collectively indicating that PLA2G4B is associated with ALD.

### 3.3 DCA-C reduces PLA2G4B expression and inhibits MAPK signaling pathway activation

**FIGURE 3:**
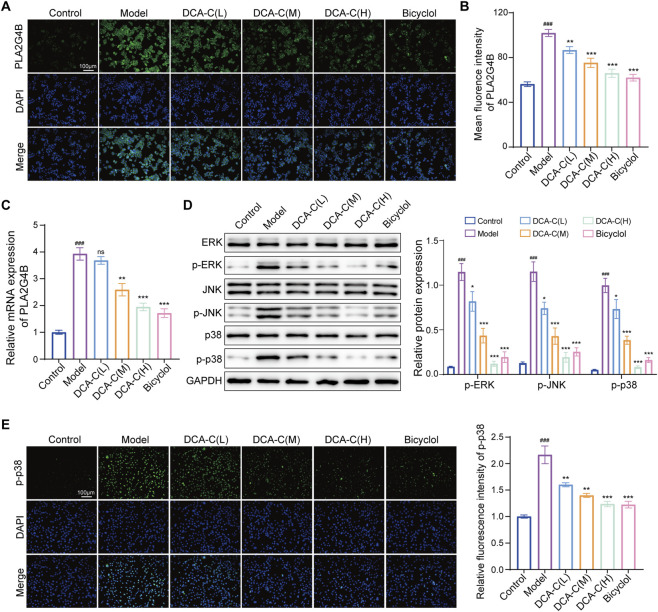
DCA-C reduces PLA2G4B expression and inhibits MAPK signaling pathway in LO2 cells. **(A,B)** Representative immunofluorescence images and quantitative analysis of PLA2G4B protein expression (scale bars: 100 μm). **(C)** PLA2G4B mRNA expression levels detected by qPCR. **(D)** Western blot analysis showing representative bands and the quantitative ratios (phosphorylated/total) of key MAPK pathway proteins. **(E)** Representative immunofluorescence images showing the subcellular localization of p-p38 (scale bars: 100 μm). Data are presented as mean ± SD (n = 3). ^###^ *p <* 0.001 vs. Control group. *p < 0.05, **p < 0.01, ***p < 0.001vs Model group.

To provide further validation for the transcriptomic results, we examined whether DCA-C could suppress the activation of the MAPK signaling pathway *in vitro*. Compared with controls, PLA2G4B expression was significantly elevated in the model group but was suppressed by DCA-C in a dose-dependent fashion, as shown by immunofluorescence and qPCR (Figures 3A–C). Notably, the reduction in PLA2G4B expression observed with the highest dose of DCA-C was comparable to that achieved by the positive control drug Bicyclol. Western blot analysis showed that the phosphorylation levels of ERK, p38, and JNK were markedly elevated in the model group, while DCA-C dose-dependently suppressed the phosphorylation of these MAPK molecules, an effect consistent with that of the positive control (Figure 3D). Additionally, immunofluorescence detection showed enhanced nuclear localization of p-p38 in the model group, which was dose-dependently attenuated by DCA-C (Figure 3E). These data indicate that DCA-C downregulates PLA2G4B expression and inhibits MAPK pathway activation in LO2 cells. The key findings were further validated in primary mouse hepatocytes. CCK-8 assays demonstrated that DCA-C treatment dose-dependently restored cell viability following ethanol-induced injury (Supplementary Figure S2A). ELISA analysis of culture supernatants revealed a dose-dependent reduction in inflammatory cytokine levels, with more pronounced effects observed at medium and high concentrations (Supplementary Figure S2B). Moreover, Western blot analysis confirmed that DCA-C treatment significantly decreased PLA2G4B protein levels in primary hepatocytes (Supplementary Figure S2C).

### 3.4 PLA2G4B overexpression compromised the ability of DCA-C to protect against alcohol-induced hepatocyte injury

**FIGURE 4:**
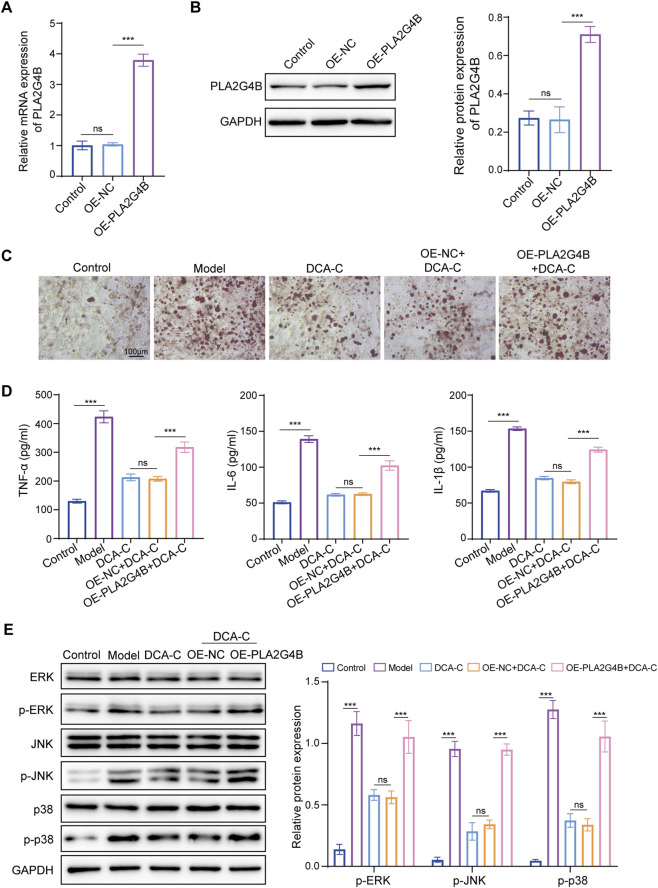
PLA2G4B overexpression compromised the ability of DCA-C to protect against alcohol-induced hepatocyte injury. **(A)** qPCR analysis of PLA2G4B mRNA expression in LO2 cells. **(B)** Western blot analysis of PLA2G4B protein expression in LO2 cells. **(C)** Representative image of LO2 cells stained with Oil red O under the different conditions. **(D)** ELISA quantification of TNF-α, IL-6 and IL-1β levels in the culture supernatant of LO2 cells under the different conditions. **(E)** Western blot analysis of the total and phosphorylated protein levels of ERK, p38, and JNK in LO2 cells. Data are presented as mean ± SD (n = 3). *p < 0.05, **p < 0.01, ***p < 0.001.

Furthermore, we assessed whether DCA-C inhibits the activation of the MAPK signaling pathway by regulating PLA2G4B. Successful overexpression of PLA2G4B in LO2 cells was confirmed at both the mRNA and protein levels by qPCR (Figure 4A) and western blot (Figure 4B), respectively. DCA-C treatment inhibited the pronounced lipid accumulation observed in the model group. However, in the OE-PLA2G4B group, this inhibitory effect was abolished, resulting in lipid deposition similar to the model group (Figure 4C). Furthermore, ELISA measurements of culture supernatants demonstrated that DCA-C significantly reduced the secretion of pro-inflammatory cytokines TNF-α, IL-6, IL-1β and this anti-inflammatory effect was also compromised by PLA2G4B overexpression (Figure 4D). Consistent with the results in LO2 cells, these findings were recapitulated in primary mouse hepatocytes (Supplementary Figures S2D,E). We next investigated the impact on the downstream MAPK pathway. Western blot analysis revealed that the phosphorylation levels of ERK, p38, and JNK were markedly elevated in the model group, while DCA-C treatment suppressed their phosphorylation, importantly, overexpression of PLA2G4B abrogated the DCA-mediated inhibition of MAPK pathway activation (Figure 4E).

### 3.5 DCA-C reduces the release of arachidonic acid by inhibiting PLA2G4B

**FIGURE 5:**
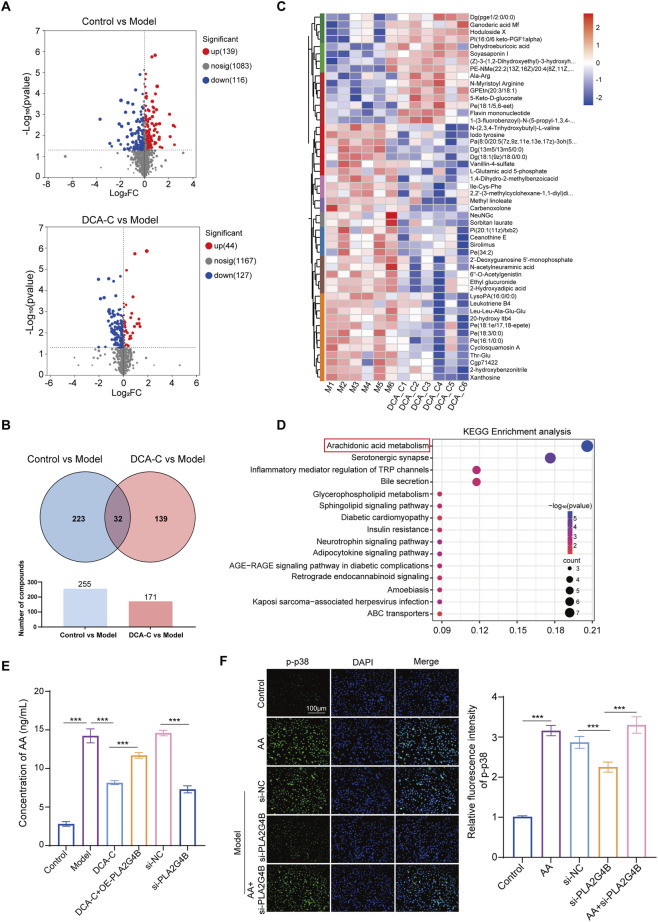
DCA-C reduces the release of arachidonic acid by inhibiting PLA2G4B. **(A)** The volcano plot illustrates the differential metabolites between the model group and the control group (top panel) as well as between the DCA-C treatment group and the model group (bottom panel). **(B)** Venn diagram. **(C)** Heatmap of differentially metabolites. **(D)** KEGG pathway enrichment analysis of differentially metabolites. **(E)** AA levels in supernatant were measured after different treatments by enzyme-linked immunosorbent assay. **(F)** Representative immunofluorescence images showing the subcellular localization of p-p38 (scale bars: 100 μm). Data are presented as mean ± SD (n = 3). ***p < 0.001.

To further investigate the downstream mechanisms through which DCA-C acts via PLA2G4B, we performed metabolomic analysis. Volcano plot analysis revealed distinct differential metabolites between the model group and the control group, as well as between the DCA-C treatment group and the model group (Figure 5A). Intersection analysis using a Venn diagram identified a set of common metabolites that were significantly altered in both comparisons (Figure 5B). A clustered heatmap further visually represented the expression of these differential metabolites across the experimental groups (Figure 5C). KEGG pathway enrichment analysis indicated that arachidonic acid metabolism was among the most significantly affected (Figure 5D). Given that PLA2G4B, as a calcium-dependent cPLA2, mediates the hydrolysis of membrane phospholipids and releases AA, we proceeded to measure AA levels in the cell supernatant. ELISA confirmed that DCA-C treatment significantly inhibited AA release compared with the model group, while overexpression of PLA2G4B under the same conditions reversed this effect and restored AA levels (Figure 5E). To clarify the relationship between AA and downstream signaling, we treated hepatocytes with exogenous AA and observed a marked increase in p-p38 immunofluorescence, confirming that AA effectively activates the p38 MAPK pathway. Conversely, knockdown of PLA2G4B in the ALD hepatocyte model attenuated this signal, whereas supplementation with exogenous AA completely reversed the inhibitory effect of si-PLA2G4B on p38 phosphorylation (Figure 5F). These findings demonstrate that AA acts as a downstream effector mediating PLA2G4B-dependent regulation of the MAPK pathway. Collectively, the above results indicate that DCA-C suppresses MAPK signaling activation by inhibiting PLA2G4B and consequently reducing AA production.

### 3.6 DCA-C alleviates ALD via the PLA2G4B-MAPK pathway *in vivo*

**FIGURE 6:**
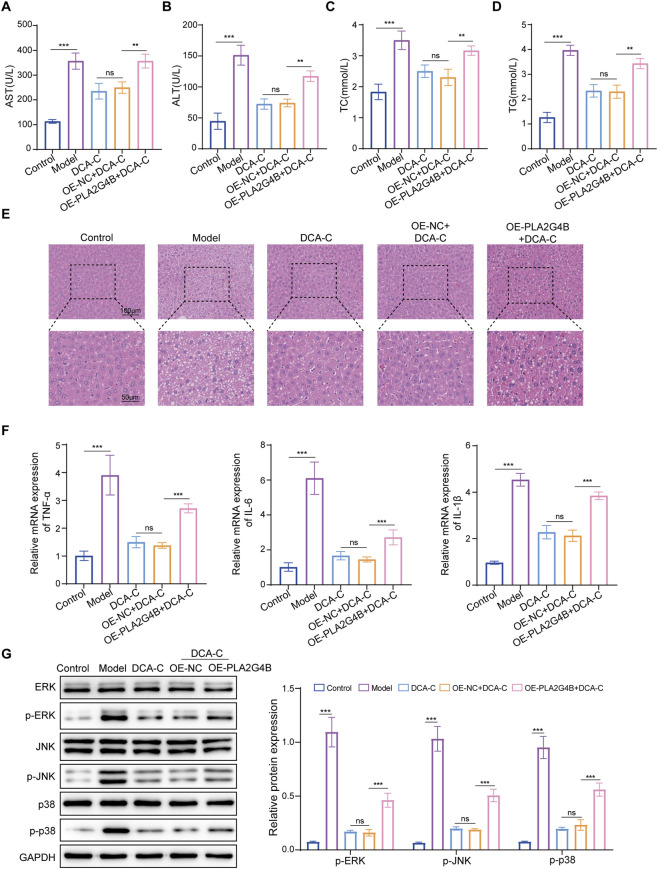
Effect of PLA2G4B overexpression on alcohol-induced liver injury in mice. **(A–C)** Serum levels of AST, ALT, and TC in mice from different treatment groups. **(D)** Hepatic triglyceride (TG) content in mouse liver tissues. **(E)** Representative photomicrographs of mouse liver sections stained with H&E. **(F)** mRNA expression levels of TNF-α, IL-6 and IL-1β in mouse liver tissues, determined by qPCR. **(G)** Western blot analysis of the phosphorylation and total protein levels of ERK, p38, and JNK in mouse liver tissues. Data are presented as mean ± SD (n = 3). *p < 0.05, **p < 0.01, ***p < 0.001.

Subsequently, we investigated whether DCA-C ameliorates ALD *in vivo* through the PLA2G4B–MAPK pathway. Serum levels of AST, ALT, and TC, which were elevated in the model group and reduced by DCA-C treatment, were significantly increased again in the OE-PLA2G4B group (Figures 6A–C). Consistent with this trend, hepatic triglyceride (TG) content, lowered by DCA-C, was also restored upon PLA2G4B overexpression (Figure 6D). H&E staining indicated that PLA2G4B overexpression counteracted the ability of DCA-C to mitigate liver injury and lipid accumulation (Figure 6E). DCA-C downregulated the mRNA expression of TNF-α, IL-6 and IL-1β, an effect that was reversed by PLA2G4B overexpression (Figure 6F). Western blot analysis confirmed that the suppression of ERK, p38, and JNK phosphorylation by DCA-C was markedly abolished in the presence of OE-PLA2G4B (Figure 6G).

## 4 Discussion

As global alcohol consumption continues to rise, alcoholic liver disease (ALD) has become a major public health issue worldwide, yet effective and safe pharmacological treatments remain limited. In recent decades, natural products have attracted increasing interest for ALD intervention due to their multi-target properties and favorable safety profiles (He et al., 2021). For instance, berberine protects against acute-on-chronic alcoholic liver injury by reshaping the gut microbiota and promoting the expansion of immunosuppressive G-MDSC-like cells through the IL6/STAT3 pathway, with its hepatoprotective activity dependent on the gut–immune axis (Li et al., 2020). Similarly, limonin, a natural limonoid, confers marked protection against both acute and chronic ethanol-induced liver damage by attenuating hepatic steatosis, oxidative stress, and inflammation (Valansa et al., 2020). Ursolic acid, a pentacyclic triterpenoid, is metabolized *in vivo* into an epoxy derivative that covalently binds to Cys-163 of caspase-3, thereby irreversibly inhibiting its activity, reducing PARP cleavage and apoptosis, and mitigating alcohol-induced liver injury in mice in a dose-dependent manner (Ma et al., 2021). Notably, these well-studied natural products primarily act on the gut–immune axis, oxidative stress, or apoptotic signaling, whereas natural compounds targeting phospholipid metabolism-driven inflammatory cascades in ALD remain largely unexplored.

In our preliminary screening of *Artemisia scoparia* extracts, we identified 4,5-dicaffeoylquinic acid (DCA-C) as a promising hepatoprotective agent. As a typical polyphenolic metabolite, DCA-C carries structural features that may confer pan-assay interference compound (PAINS) properties, which often lead to non-specific bioactivity and false-positive outcomes in conventional *in vitro* high-throughput screening. To address this critical limitation, we implemented a rigorous experimental design to minimize and exclude non-specific interference. *In vitro* assays were combined with cell morphological observation, multi-dimensional functional validation, and stringent control groups, allowing us to rule out cytotoxic artifacts, off-target interference, and PAINS-related false-positive signals and ensure the authenticity of our observations (Bolz et al., 2021).

To further validate the specific hepatoprotective effects of DCA-C *in vivo*, we employed the well-recognized NIAAA mouse model of ALD, which faithfully recapitulates early pathological features of alcoholic hepatitis and hepatic steatosis. Mice were orally administered DCA-C at 10, 30, and 100 mg/kg daily for 10 consecutive days starting from the first day of ethanol feeding. Our results demonstrated that DCA-C dose-dependently alleviated alcohol-induced liver injury, as reflected by reduced serum levels of ALT, AST, TC, and TG, attenuated hepatic lipid accumulation, and suppressed pro-inflammatory cytokine expression. The protective effect of 100 mg/kg DCA-C was comparable to that of the positive control drug bicyclol at 200 mg/kg. Collectively, these *in vivo* findings firmly confirm that the observed bioactivity represents specific pharmacological action rather than non-specific effects caused by PAINS-like characteristics. Furthermore, the hepatoprotective efficacy of DCA-C is highly consistent with the traditional ethnopharmacological use of *A.rtemisia scoparia* for liver disorders, reinforcing the reliability and rationality of our findings.

Mechanistically, transcriptomic profiling identified PLA2G4B as a potential target of DCA-C, and subsequent experiments verified that DCA-C directly binds to PLA2G4B and downregulates its protein expression. Metabolomic analysis further revealed arachidonic acid (AA) as a key downstream metabolite in this regulatory axis. As a calcium-dependent cytosolic phospholipase A2, PLA2G4B catalyzes the hydrolysis of arachidonoyl phospholipids to release AA (Cai et al., 2025). On the basis of these results, we conclude that DCA-C inhibits the excessive release of AA by directly targeting PLA2G4B, thereby blocking the hyperactivation of the MAPK signaling pathway and ultimately relieving ethanol-induced hepatic steatosis and inflammation. Our study thus systematically elucidates the molecular basis underlying the anti-steatotic and anti-inflammatory effects of DCA-C, establishing a clear regulatory network for the targeted treatment of ALD.

Hepatic steatosis is a complex pathological process governed by multiple interconnected signaling networks. Wang et al. reported that Akt2 and AMPKα2 double knockout mitigates high-fat diet-induced obesity and hepatic steatosis by enhancing Parkin-mediated mitophagy, indicating that defective mitophagy and dysregulated metabolic kinases contribute substantially to abnormal hepatic lipid deposition (Wang et al., 2021). Dusabimana et al. demonstrated that P2Y2R deficiency attenuates high-fat diet-induced steatosis by activating AMPK/PGC-1α to boost mitochondrial fatty acid β-oxidation (Dusabimana et al., 2021). In addition, activation of the AMPK–SREBP-1c pathway suppresses *de novo* lipogenesis and alleviates hepatic steatosis in models of alcoholic fatty liver disease (Wan et al., 2024). In contrast, our study uncovered a distinct mechanism by which DCA-C protects against alcoholic hepatic steatosis: instead of modulating mitophagy, fatty acid β-oxidation, or *de novo* lipogenesis, DCA-C directly targets PLA2G4B to reduce AA production and inhibit MAPK overactivation. This regulatory axis has rarely been reported for natural products used in ALD therapy, distinguishing DCA-C from agents that act on mitochondrial function, energy sensing, or lipogenic pathways.

Several limitations of this study should be acknowledged. First, similar to many natural products, DCA-C may exhibit multi-target binding properties. Although cellular thermal shift assay (CETSA) and molecular docking data support the direct interaction between DCA-C and PLA2G4B, we cannot exclude the possibility that DCA-C binds additional targets that contribute to its overall hepatoprotective effects. This promiscuity makes it difficult to definitively attribute the therapeutic efficacy solely to PLA2G4B inhibition, and potential off-target effects warrant further investigation. Second, target selectivity remains to be fully characterized. Given the high structural conservation of the catalytic domains among PLA2 family members, whether DCA-C selectively inhibits PLA2G4B without affecting other phospholipases requires further validation. Third, the present study only focused on early-stage alcohol-induced liver injury and steatosis and did not address advanced pathological stages such as liver fibrosis and cirrhosis. Future studies using PLA2G4B-specific knockout models, comprehensive target profiling, and assessment in fibrotic chronic ALD models will be necessary to fully validate the therapeutic potential and target specificity of DCA-C for ALD.

## Data Availability

The data that support the findings of this study are available from the corresponding author upon reasonable request.
