# Supplementary figures and images for: Dicaffeoylquinic acid alleviates alcoholic liver disease by targeting PLA2G4B and inhibiting the MAPK signaling pathway
Source: Front Pharmacol. 2026 May 29;17:1823992. doi: 10.3389/fphar.2026.1823992 (PMC13259897; doi:10.3389/fphar.2026.1823992)

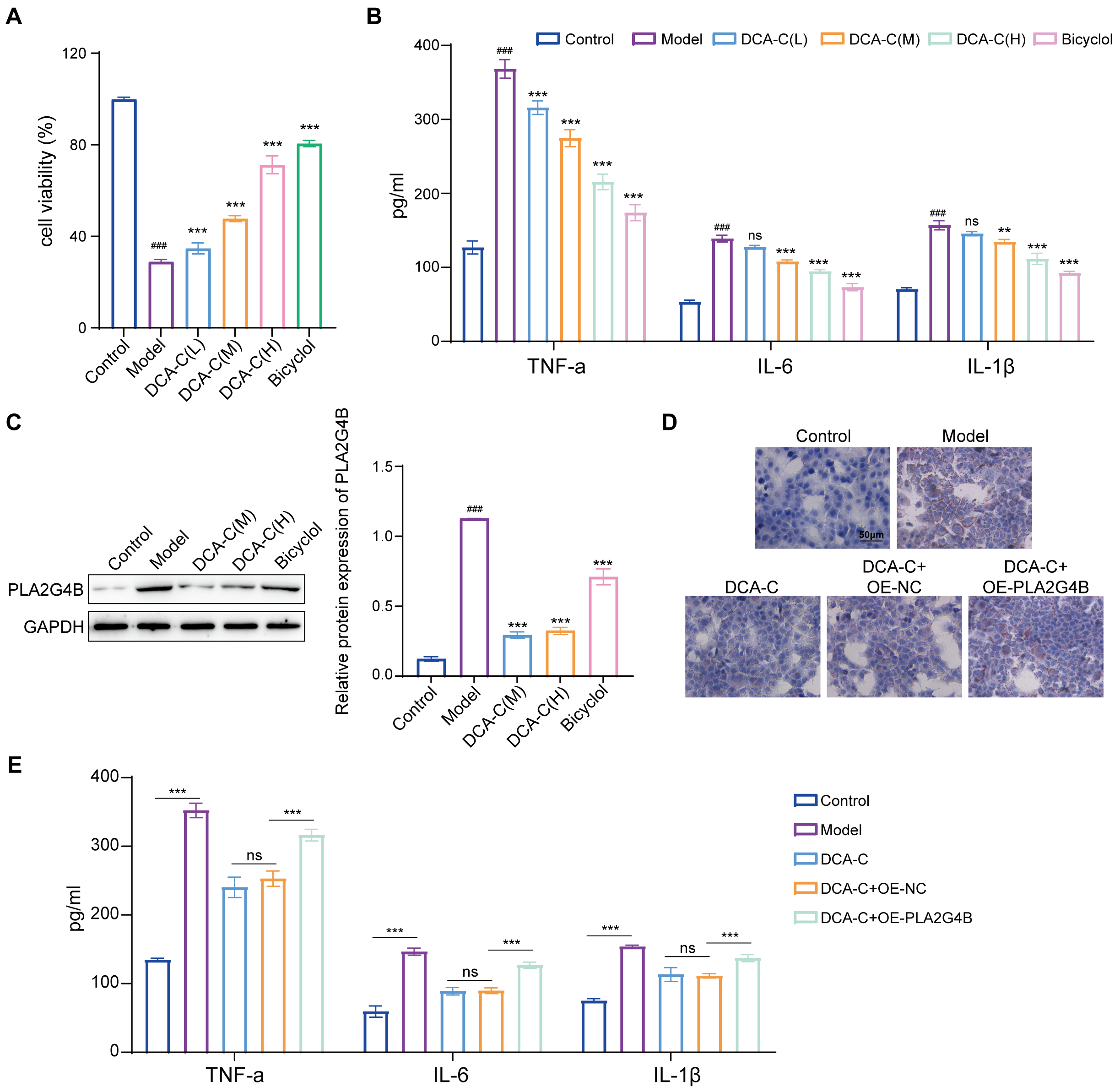

Supplement: Supplementary file 1 [file Image2.tif]

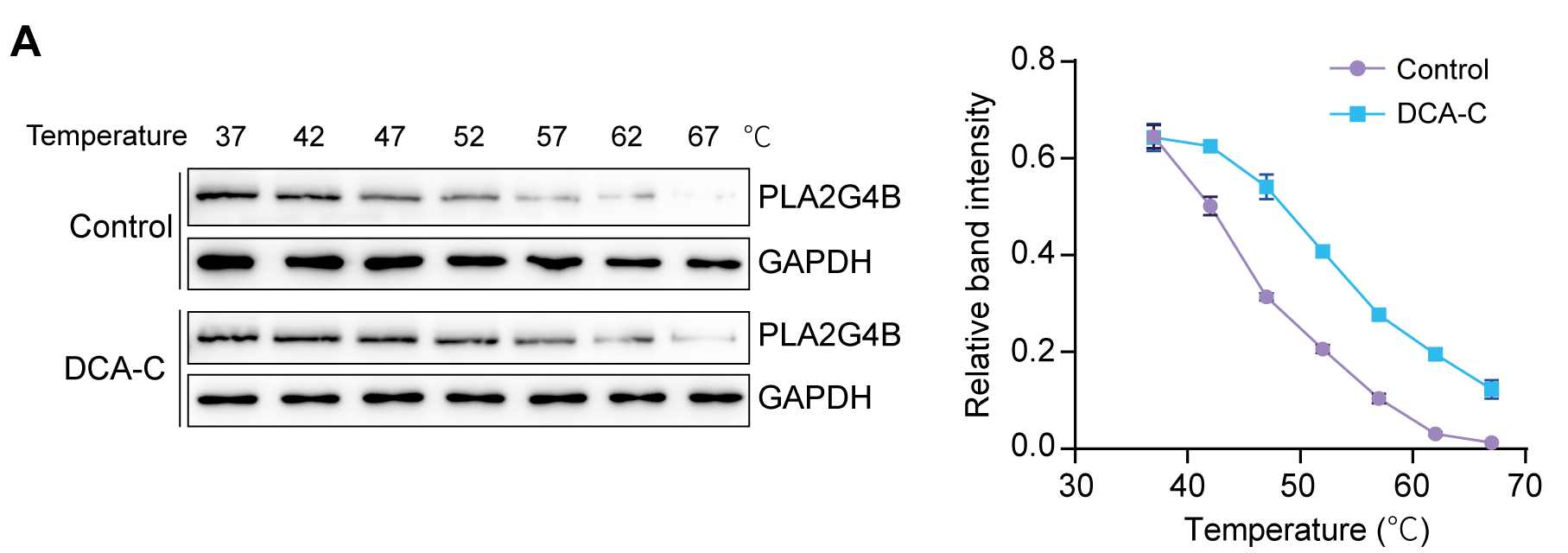

Supplement: Supplementary file 2 [file Image1.tif]
