# Supplementary material for: Dicaffeoylquinic acid alleviates alcoholic liver disease by targeting PLA2G4B and inhibiting the MAPK signaling pathway
Source: Front Pharmacol. 2026 May 29;17:1823992. doi: 10.3389/fphar.2026.1823992 (PMC13259897; doi:10.3389/fphar.2026.1823992)
Supplement: Supplementary file 3 [file DataSheet1.pdf]

Supplementary Figure 1

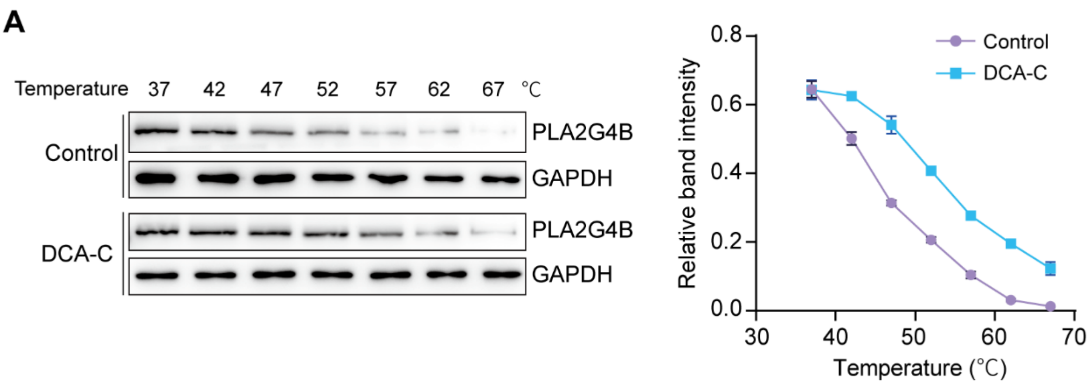

The cell thermal migration experiment verified the binding characteristics of DCA-C with PLA2G4B.

Supplementary Figure 2

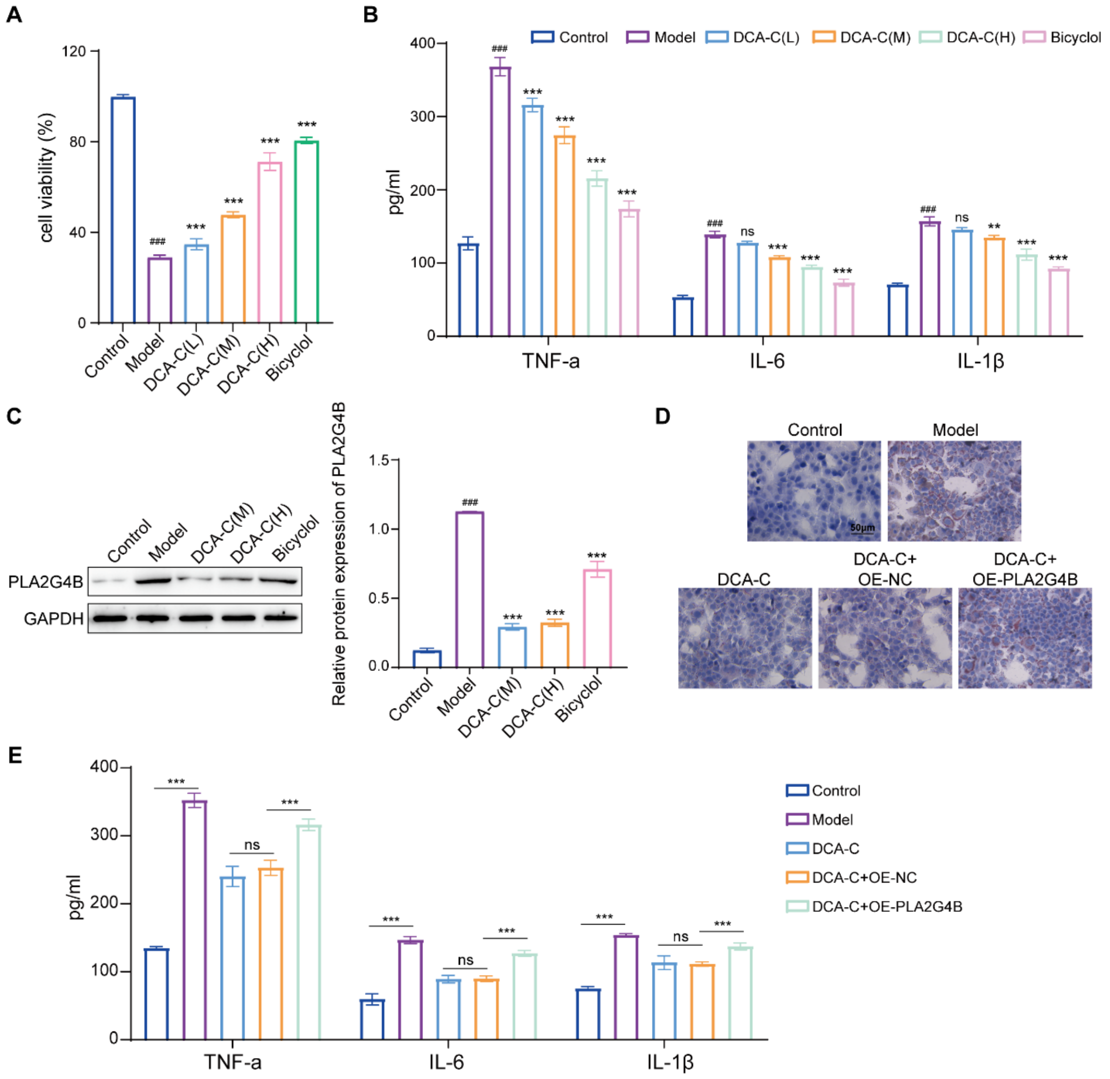

DCA-C reduces PLA2G4B expression in primary mouse hepatocytes. (A) Cell

viability measured by CCK-8 assay. (B) ELISA quantification of TNF-α, IL-6 and IL-1β levels in the culture supernatant of primary mouse hepatocytes under the different conditions. (C) Western blot analysis of PLA2G4B protein expression. (D) Representative image of primary mouse hepatocytes stained with Oil red O under the different conditions (scale bars: 50 μm). (E) ELISA quantification of TNF-α, IL-6 and IL-1β levels in the culture supernatant of primary mouse hepatocytes under the different conditions. Data are presented as mean ± SD (n=3). $^{###}p < 0.001$ vs Control group. $^{*}p < 0.05$, $^{**}p < 0.01$, $^{***}p < 0.001$vs Model group.
